# Associations of retinal neurovascular dysfunction with inner retinal layer thickness in non-proliferative diabetic retinopathy

**DOI:** 10.1007/s00417-024-06552-4

**Published:** 2024-06-15

**Authors:** Berthold Pemp, Stefan Palkovits, Stefan Sacu, Doreen Schmidl, Gerhard Garhöfer, Leopold Schmetterer, Ursula Schmidt-Erfurth

**Affiliations:** 1 Department of Ophthalmology, Medical University of Vienna, Währinger Gürtel 18-20, Vienna, 1090 Austria; 2 Department of Clinical Pharmacology, Medical University of Vienna, Vienna, Austria; 3 Department of Ophthalmology, Hanusch Hospital Vienna, Vienna, Austria; 4 Singapore Eye Research Institute, Singapore, Singapore; 5 National University of Singapore, Duke-NUS Medical School, Singapore, Singapore; 6 School of Chemical and Biological Engineering, Nanyang Technological University, Singapore, Singapore

**Keywords:** Diabetes mellitus, Diabetic retinopathy, Neurovascular coupling, Retinal neurodegeneration, Optical coherence tomography, Dynamic vessel analysis

## Abstract

**Purpose:**

Neurovascular coupling impairment and inner retinal layer thinning are early detectable retinal changes in diabetes, and both worsen during progression of diabetic retinopathy (DR). However, direct interactions between these features have not been investigated so far. Therefore, we aimed to analyze associations between the retinal functional hyperemic response to light stimulation and the thickness of individual neuroretinal layers in eyes with early non-proliferative DR.

**Methods:**

Thirty patients with type 1 diabetes featuring mild (*n* = 15) or moderate (*n* = 15) non-proliferative DR and 14 healthy subjects were included in this cross-sectional study. Retinal vessel diameters were measured before and during illumination with flickering light using a dynamic vessel analyzer. Individual layer thickness in the macula was analyzed from spectral domain optical coherence tomography.

**Results:**

Flicker light-induced vessel dilation was significantly reduced in patients compared to healthy controls (veins: 3.0% vs. 6.1%, *p* < 0.001; arteries: 1.3% vs. 5.1%, *p* = 0.005). Univariately, the response in retinal veins of diabetes patients correlated significantly with ganglion cell layer (GCL) thickness (*r* = 0.46, *p* = 0.010), and negatively with hemoglobin A1c (HbA1c) levels (*r*=-0.41, *p* = 0.023) and age (*r*=-0.38, *p* = 0.037), but not with baseline diameters, glucose levels, or diabetes duration. In a multiple regression model only GCL thickness (*p* = 0.017, β = 0.42) and HbA1c (*p* = 0.045, β=-0.35) remained significantly associated with the vascular flicker light response.

**Conclusion:**

The results indicate that thinner GCL and worse glycemic control both contribute to reduced retinal neurovascular coupling in patients with clinical signs of DR. Progression of neurovascular dysfunction in DR might be related to structural degeneration of the neurovascular complex in the inner retina.

## Key message

***What is known:***

• Neurovascular coupling dysfunction and neurodegeneration in the inner retina are early features in eyes of diabetes patients, and both worsen during progression of diabetic retinopathy. However, direct interactions between these changes have not been investigated so far.

***What is new:***

• Our study shows that in patients with early diabetic retinopathy the reduced functional hyperemic response to flicker light stimulation correlates significantly with inner retinal layer thickness, and concurrently with worse glycemic control.

• This demonstrates for the first time that retinal neurodegeneration may be an important contributor to the advancing disturbance of neurovascular coupling in eyes with clinically established diabetic retinopathy.

## Introduction

Clinical diagnosis and staging of diabetic retinopathy (DR) is based on the characteristic microvascular changes in the retina observed by biomicroscopy, fundus photography and fluorescein angiography. However, more advanced techniques in ocular imaging also enable to study subtle changes in retinal microanatomy as well as perfusion alterations in vivo, that otherwise remain clinically unnoticed. Degenerative changes in both the neural and the vascular components of the retina have been demonstrated as early features in experimental diabetes and it is often debated which one is affected first in DR [1].

An increasing number of clinical studies using optical coherence tomography (OCT) indicates that changes in DR include the degeneration of neuroretinal tissue, which has been described as diabetic neuroretinopathy [2–6]. This degeneration is seen mostly in the inner retinal layers of the perifoveolar region [6–11], where these layers are thickest, and it worsens with advancing stages of DR [12, 13]. In line with these more recent findings, multiple studies have demonstrated subclinical impairment of neuroretinal function in patients with early DR, and on a smaller scale also in diabetes patients without DR [14–22]. In addition, reduced capillary density in the fovea can be observed early on using fluorescein angiography or OCT angiography [23, 24]. It worsens in correlation with inner retinal layer thinning during progression of DR [13, 25–27], and leads to reduced capillary blood flow in neuroretinal tissue [28, 29].

Blood flow in the retina is locally controlled by autoregulation, which enables a constant blood supply during changes in perfusion pressure [30]. In addition, when neuronal cells in the retina are activated, their metabolic and blood demand increase. This is compensated by a regulatory increase in blood flow, a response termed neurovascular coupling or functional hyperemia [31, 32]. This response is mediated by signaling through glial cells that are in direct contact to neurons and vessel walls. Due to the tight interaction of neurons, glia and pericytes in the regulation of neurovascular coupling, these three cell types can collectively be termed as the neurovascular complex. Accordingly, both vascular and neuronal alterations in the diabetic retina could play a part in the disturbed blood flow response to retinal stimulation that has been observed in many studies [33]. Stimuli suitable for assessing neurovascular coupling in the retina include transient exposure to flickering light or the transition from dark-adapted state to light. Both stimuli are known to increase blood flow and vessel diameters in the healthy retina, which can be measured non-invasively using various imaging devices [33].

Hyperglycemia significantly affects vascular reactivity to retinal stimulation already in healthy eyes [34, 35], and impaired neurovascular coupling is also among the earliest detectable changes in the retina of diabetes patients [36–38]. Interestingly, this alteration has been found associated with but also independent from retinal ganglion cell dysfunction [39, 40]. It has also been shown that neurovascular coupling deteriorates further with increasing stages of DR [37, 41, 42]. However, the influence of neurodegenerative changes in this context is not known.

To our knowledge, direct interactions between retinal layer structure and the disturbed neurovascular coupling in DR have not been investigated so far. Therefore, we aimed to examine associations of the functional hyperemic response in the retina during flicker light stimulation with the thickness of the individual neuroretinal layers in patients with early non-proliferative DR, in whom retinal layer thinning might be expected.

## Materials and methods

The study was performed at the Department of Ophthalmology and the Department of Clinical Pharmacology of the Medical University of Vienna in adherence to the tenets of the Declaration of Helsinki and Good Clinical Practice guidelines. The study protocol and its design was approved by the institutional Ethics Committee. All participants signed written informed consent before the study.

### Study participants

Thirty adult patients with type 1 diabetes featuring mild (*n* = 15) or moderate (*n* = 15) non-proliferative DR and 14 healthy subjects registered as probands at the Department of Clinical Pharmacology and matched for sex and age were included. The sample size of this study was specified to detect differences in vessel diameters and vessel reactivity between groups, as observed in previous experiments [36, 39, 40]. A sample size of 30 patients was estimated sufficient for exploratory correlation analysis. All participants had a screening examination including detailed medical history, a complete ophthalmologic examination, and assessment of best-corrected visual acuity (BCVA) using standardized logarithmic visual acuity charts (“ETDRS” Charts, Precision Vision, La Salle, IL). Resting blood pressure was measured and venous blood samples were taken to confirm normal blood pressure, blood count and glucose levels in healthy individuals. Hemoglobin A1c (HbA1c) was quantified in diabetes patients but not in healthy controls. DR was classified according to the criteria set out in the Early Treatment Diabetic Retinopathy Study (ETDRS) [43]. Patients without DR or with more advanced stages than moderate DR, patients after a previous treatment with intravitreal injections or laser and patients with macular oedema were not included. Further exclusion criteria for all participants were or consumption of illicit drugs or nicotine products including tobacco smoking, a body mass index above 30 kg/m², other eye diseases, anti-inflammatory medication in the past 3 weeks, and refractive errors of more than 6 diopters. If both eyes had DR, the eye showing more signs of DR was chosen as the study eye. In diabetes patients capillary blood glucose was measured during the study day. All participants had to refrain from consuming alcohol or caffeine during 12 h before the study.

### Dynamic vessel analyzer

Study eyes were instilled with tropicamide for pupil dilation. After a resting period of 15 min, we measured the diameters of one major temporal retinal artery and vein supplying the macular area, at a location up to two disc diameters from the optic disc margin, using the Dynamic Vessel Analyzer (DVA, IMEDOS GmbH, Jena, Germany). The DVA is described in detail elsewhere [44]. In short, it enables continuous real-time analysis of retinal vessel diameters from digitized images. Baseline measurements were performed for 60 s. This was followed by 60 s of stimulation with full field flickering light at a frequency of 12.5 Hz, during which the response of vessel diameters could be assessed. Flicker light-induced vasodilation was defined as the ratio of the average diameter values during the last 20 s of the stimulation period over the average values during the whole baseline recording, and was expressed as % change over baseline. Measurement accuracy of the DVA is higher in retinal veins [44]. Therefore, the venous response was chosen as the primary outcome variable of vessel reactivity.

### Optical coherence tomography

Spectral domain OCT (Spectralis OCT, Heidelberg Engineering, Heidelberg, Germany) was performed using a 20 × 20 degree scan pattern of 49 horizontal B-scans centered on the macula. Technical properties of this OCT system are detailed elsewhere [45]. Activated eye-tracking with averaging of 20 frames per B-scan was used to minimize motion artefacts and background noise, and to enhance scan contrast [46]. The built-in segmentation software (HRA viewing module version 6.7.17; Heidelberg Engineering) was used for automated segmentation of individual retinal layers. All B-scans were checked for segmentation errors by the same investigator (BP), and obvious errors were manually corrected. Thus, three-dimensional maps of retinal nerve fiber layer, ganglion cell layer (GCL), inner plexiform layer (IPL), inner nuclear layer, outer plexiform layer, and outer nuclear layer were obtained. A circular ETDRS grid was centered on the foveola, and the average layer thicknesses in the ring areas measuring 1–3 mm, and 3–6 mm around the center were used for quantitative analysis. The inner ring area was chosen as primary outcome variable of retinal layer thickness, because it contains the maximum thickness of GCL and IPL [9].

### Statistical analysis

Continuous variables were expressed as means ± standard deviation (SD). Normal distribution was assessed by the Shapiro-Wilk test and accepted at *p* > 0.05. Unpaired t-tests were used to compare normally distributed data between groups. Diameter changes in retinal veins were assessed by analysis of covariance (ANCOVA) using baseline vessel diameters as a co-variable. Categorical variables and non-normally distributed data were compared using the Mann-Whitney U test. Univariate analyses with Pearson correlations were calculated to assess correlations of flicker light response or retinal layer thickness with other variables. In addition, multiple regression models with flicker light response or retinal layer thickness as dependent variables were performed. A p-value of 0.05 was considered as the level of significance for all calculations. The Statistica software (Release 6.1; StatSoft Inc., Tulsa, OK) was used for all statistical analyses.

## Results

### Subject characteristics and study parameters

Table 1 displays the characteristics and measurement results in the groups of investigated patients and healthy controls. The primary outcome variables were normally distributed in all groups.

**Table 1:** Summarized data of healthy control subjects and diabetes patients, and of patient subgroups

	Healthy controls (*n* = 14)	Diabetes patients (*n* = 30)	*P* values	Mild retinopathy (*n* = 15)	Moderate retinopathy (*n* = 15)	*P* values
Age (years)	40 ± 20	46 ± 11	0.055^†^	43 ± 9	50 ± 13	0.100
Sex (male/female)	9 / 5	20 / 10	0.970^†^	8 / 7	11 / 4	0.367^†^
Height (cm)	175 ± 8	172 ± 7	0.342	172 ± 8	172 ± 8	0.905
Weight (kg)	74 ± 14	73 ± 12	0.906	75 ± 16	72 ± 8	0.632
Disease duration (years)	-	29 ± 9	-	26 ± 9	31 ± 8	0.163
Glucose level (mg/dL)	-	144 ± 62	-	147 ± 60	142 ± 65	0.822
Hemoglobin A1c (%)	-	7.6 ± 1.1	-	7.8 ± 1.1	7.4 ± 1.1	0.235
Visual acuity (logMAR)	-0.06 ± 0.11	-0.04 ± 0.06	0.306^†^	-0.05 ± 0.05	-0.04 ± 0.08	0.820^†^
Arterial diameter (µm)	132 ± 17	123 ± 18	0.099^†^	126 ± 14	119 ± 21	0.443^†^
Venous diameter (µm)	155 ± 17	159 ± 20	0.772^†^	157 ± 19	161 ± 22	0.552
Arterial flicker light response (%)	5.1 ± 5.2	1.3 ± 2.3	**0.005** ^†^	1.1 ± 2.5	1.4 ± 2.1	0.539^†^
Venous flicker light response (%)	6.1 ± 3.1	3.0 ± 2.3	< 0.001*	2.7 ± 1.9	3.3 ± 2.7	0.562*
Retinal nerve fiber layer thickness (µm)	21.9 ± 1.2	22.8 ± 2.5	0.184	23.3 ± 4.6	22.4 ± 3.2	0.357
Ganglion cell layer thickness (µm)	50.7 ± 3.3	50.1 ± 7.1	0.782	51.6 ± 5.9	48.7 ± 8.1	0.284
Inner plexiform layer thickness (µm)	41.4 ± 4.1	40.6 ± 4.9	0.650^†^	41.7 ± 3.9	39.5 ± 5.7	0.236
Inner nuclear layer thickness (µm)	39.0 ± 2.6	39.2 ± 4.1	0.846	39.7 ± 3.8	38.8 ± 4.5	0.561
Outer plexiform layer thickness (µm)	33.4 ± 3.4	34.0 ± 3.3	0.558	33.5 ± 3.3	34.6 ± 3.4	0.376
Outer nuclear layer thickness (µm)	73.0 ± 5.7	69.7 ± 9.1	0.115^†^	71.4 ± 9.3	68.1 ± 8.9	0.342

Data are presented as means ± SD. Bold values indicate significant p-values in group comparisons (*p* < 0.05); unpaired t-test for normally distributed data, except * = ANCOVA using baseline diameter as co-variable; † = Mann-Whitney U test for non-normally distributed data

Age, sex, height, weight, visual acuity and vessel diameters were not significantly different between patients and healthy controls. In addition, all parameters in the two patient subgroups with mild or moderate DR did not differ statistically. Besides daily insulin, 20 patients had additional regular blood pressure and/or lipid-lowering medication including angiotensin-converting-enzyme inhibitors, angiotensin II receptor blockers, beta blockers and statins.

### Vessel diameter changes

**Fig. 1:**
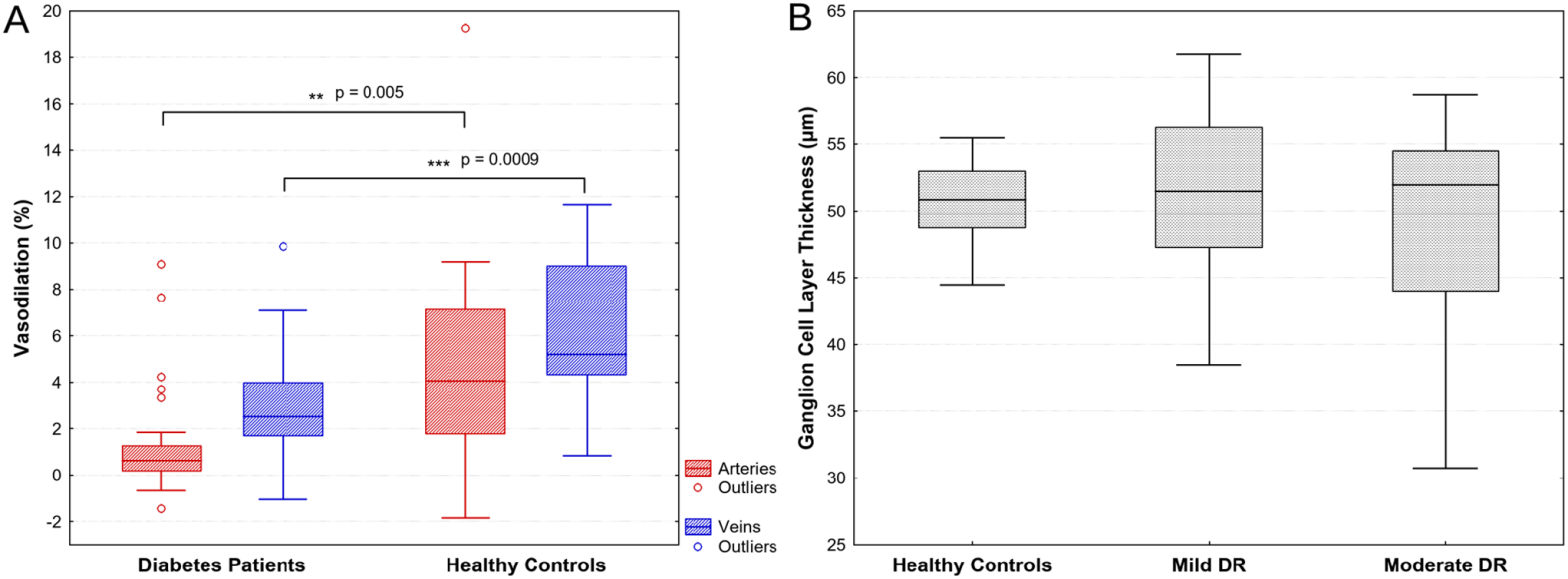
Box-and-whisker plots presenting retinal vessel dilation in response to stimulation with flickering light (**A**) and macular ganglion cell layer thickness (**B**) in patients with diabetic retinopathy (DR) and in healthy controls. Data are presented as median (bar), interquartile range (box), non-outlier minimum and maximum (whiskers) and outliers (dots)

Vessel dilation in response to stimulation with flickering light was significantly reduced in the diabetes patients compared to healthy controls in retinal veins (3.0%±2.3% vs. 6.1%±3.1%, *p* < 0.001; Fig. 1A) as well as in retinal arteries (1.3%±2.3% vs. 5.1%±5.2%, *p* = 0.005). The flicker light response was neither significantly different in patients with mild or moderate DR (Table 1) nor in patients with or without co-medication (veins: 2.7%±2.1% vs. 3.6%±2.6%, *p* = 0.35; arteries: 1.4%±2.5% vs. 1.0%±1.8%, *p* = 0.69).

**Fig. 2:**
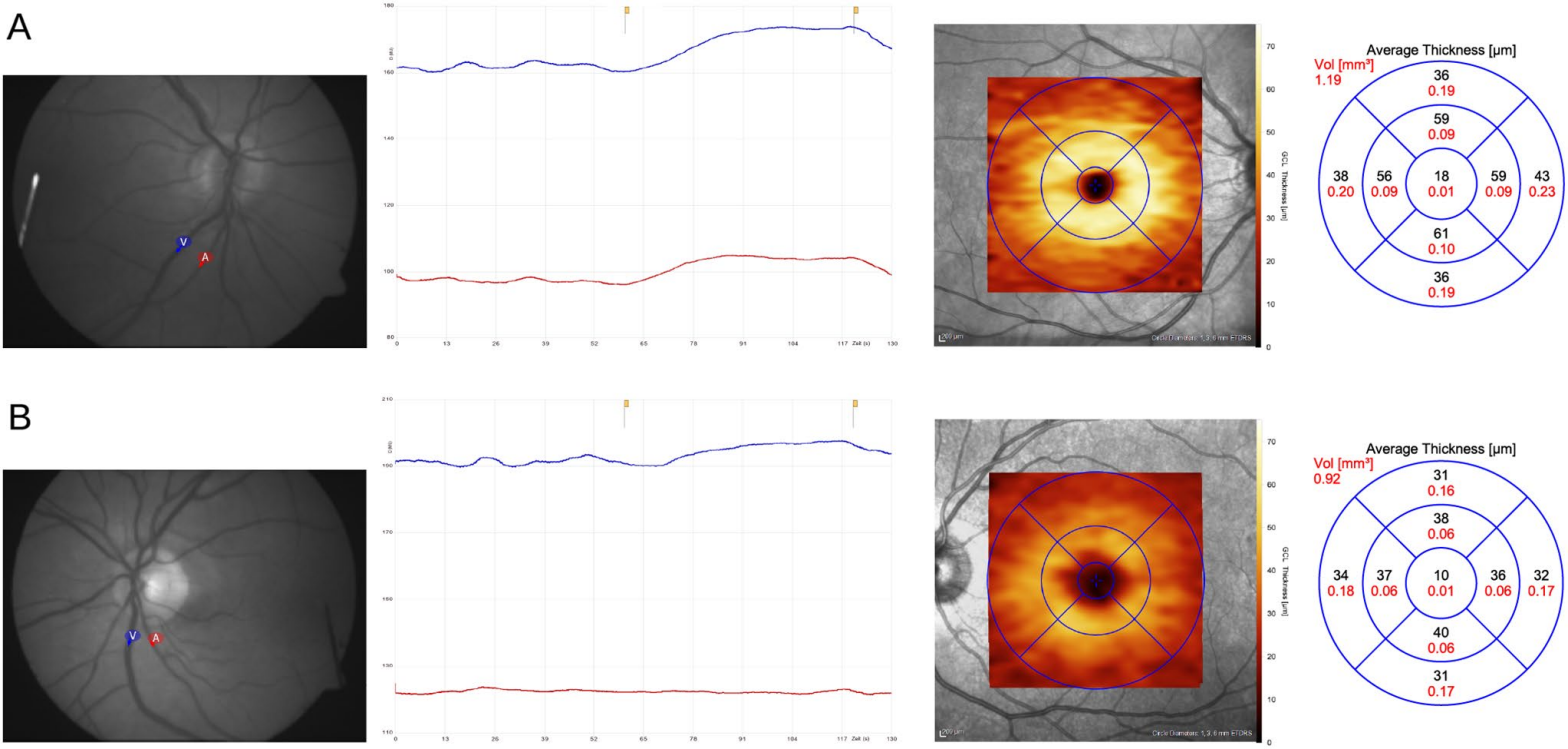
Vessel diameters during the flicker light experiment using the DVA, and macular ganglion cell layer thickness heatmaps and data from OCT in two diabetes patients without (**A**) and with (**B**) diabetic neuroretinopathy. Blue and red curves represent venous and arterial diameters, respectively; yellow marks indicate beginning and end of the stimulation period. The thickness heatmap is overlaid with a 6 mm ETDRS grid [43]. Average thickness in the individual sectors is shown in the adjacent diagram

DVA and OCT measurements in two exemplary diabetes patients are shown in Fig. 2.

### Correlation analyses

Table 2 summarizes the results of univariate correlation analyses between the venous flicker light response and individual retinal layer thickness in diabetes patients.

**Table 2:** Summary of univariate correlation analyses between venous flicker light response and individual retinal layer thickness as measured by OCT in diabetes patients

Retinal layer	ETDRS grid area	*r*	*P* values
Retinal nerve fiber layer	1–3 mm ring area	0.17	0.372
	3–6 mm ring area	0.12	0.522
Ganglion cell layer	1–3 mm ring area	0.46	**0.010**
	3–6 mm ring area	0.29	0.116
Inner plexiform layer	1–3 mm ring area	0.40	**0.027**
	3–6 mm ring area	0.27	0.144
Inner nuclear layer	1–3 mm ring area	0.34	0.065
	3–6 mm ring area	0.12	0.532
Outer plexiform layer	1–3 mm ring area	0.05	0.777
	3–6 mm ring area	-0.08	0.658
Outer nuclear layer	1–3 mm ring area	-0.08	0.692
	3–6 mm ring area	-0.11	0.554

Bold values indicate significant p-values (*p* < 0.05); r = Pearson’s correlation coefficient

**Fig. 3:**
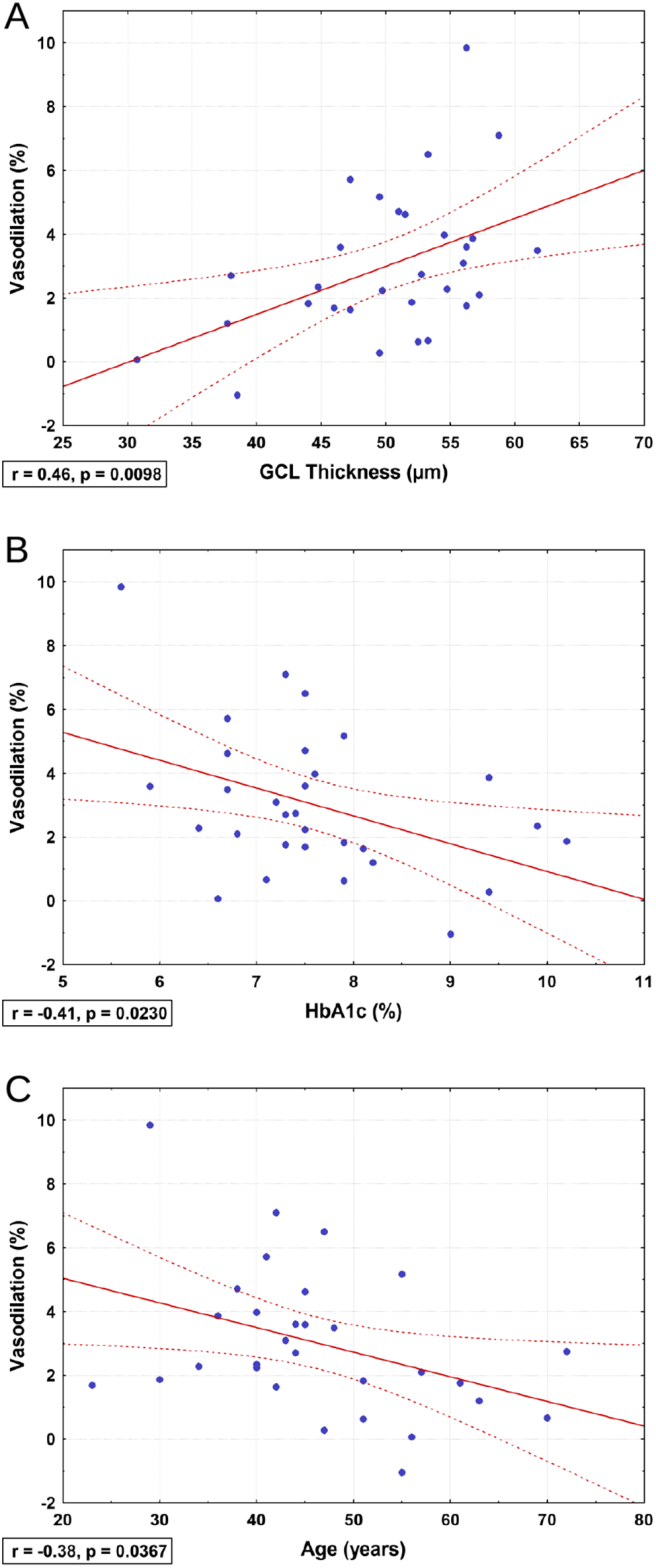
Scatterplots with correlation analyses of the flicker light response in retinal veins with ganglion cell layer (GCL) thickness (**A**), hemoglobin A1c (HbA1c) levels (**B**) and age (**C**) in diabetes patients. Straight lines show linear fits, and dotted lines show 0.95 confidence intervals

In diabetes patients only, univariate analysis showed a significant correlation of the diminished flicker light response in retinal veins with GCL thickness (*r* = 0.46, *p* = 0.010; Fig. 3A) and to a lesser degree with IPL thickness (*r* = 0.40, *p* = 0.027), whereas all other retinal layer thickness values did not correlate. In addition, the venous flicker light response correlated negatively with HbA1c levels (*r*=-0.41, *p* = 0.023; Fig. 3B) and with age (*r*=-0.38, *p* = 0.037; Fig. 3C), but not with baseline vessel diameter (*r* = 0.08, *p* = 0.69), glucose level (*r* = 0.00, *p* = 0.98), or diabetes duration (*r*=-0.27, *p* = 0.15). In a multiple regression model including GCL thickness, age, disease duration, baseline venous diameter, blood glucose, HbA1c and arterial hypertension as predictor variables, only GCL thickness (*p* = 0.017, β = 0.42) and HbA1c (*p* = 0.045, β=-0.35) remained significantly associated with the vascular flicker light response.

Mean GCL thickness was slightly but not significantly lower in diabetes patients, more obvious in patients with moderate DR (Table 1). Figure 1B illustrates a more negative skew of GCL thickness values in eyes with moderate DR. GCL thickness did neither show significant correlations with age, diabetes duration or HbA1c in univariate analysis nor in a multivariate model. Including DR stage as an additional categorical factor also showed no correlations.

In healthy control subjects no significant association between flicker light-induced vasodilation with any other variable, including GCL thickness (*r* = 0.28, *p* = 0.34) and age (*r*=-0.20, *p* = 0.50), was detected. Interaction analysis of vessel reactivity in the combined groups showed a statistically significant interaction term of group and GCL thickness (*p* = 0.036) and an increase in r² (0.36 vs. 0.13), which indicates an effect of GCL thickness on vasodilation in diabetes patients.

## Discussion

Our results demonstrate an association of the impaired flicker light-induced hyperemic response in eyes with early DR with the gradual reduction of inner retinal layer thickness, most prominently in the macular GCL. Not all patients were equally affected by GCL thinning. Hence, GCL thickness was not significantly different between the study groups. However, its significant correlation with retinal vessel reactivity in the diabetes patients indicates a neurodegenerative component in the worsening of neurovascular coupling in eyes with DR.

The results are in good agreement with previous OCT studies in diabetes that found subtle atrophic changes primarily in the inner retinal layers [3–12, 47]. Recent longitudinal observations have shown a progressive GCL thinning already before clinical emergence of DR [13, 48, 49]. Long-term follow-up revealed a mean parafoveal GCL + IPL loss of 0.29 to 0.46 μm per year, independent of HbA1c, age, and sex [6, 48]. Apoptosis of neuroretinal cells and GCL reduction are early and persistent features in diabetes [1, 6, 50–53]. However, since the GCL contains retinal ganglion cells, Müller cells, astrocytes, microglia, displaced amacrine cells, and capillaries of the superficial retinal vascular plexus [11], layer thinning may not only involve ganglion cell degeneration but also glial and microvascular loss.

Obviously, not all patients are affected by diabetic neuroretinopathy to a similar extent. The reason for this is not clear. Despite a relatively long disease duration in our cohort we detected no association of GCL thinning with disease duration or medium-term glycemic control. In addition, there was no significant difference in retinal layer thickness between the two subgroups of non-proliferative DR. Patients with moderate DR tended to have longer disease duration and lower GCL thickness. However, a considerable variability of these parameters is noted in both patient subgroups. The sample size may have been too small to detect retinal layer thinning on a group level as previously shown by others.

It has been speculated that retinal neurodegeneration in diabetes may play a role in the formation of early microvascular changes in DR, including breakdown of the blood-retina barrier, vasoregression and impairment of neurovascular coupling [4]. However, the causal relationship of neuroretinal degeneration and diabetic microangiopathy has not been clarified. Subclinical dysregulation and changes in the microcirculation could also induce or enhance neurodegenerative processes via an increased vulnerability to damage from metabolic injury.

In line with our results, multiple studies in diabetes patients have shown a reduction of vessel reactivity to flickering light, with comparable values in mild and moderate DR [36, 37, 39, 41], indicating abnormal neurovascular coupling. This impairment can often be observed already before the first visible signs of DR [33, 40, 54], and to a lesser extent in pre-diabetic patients [55] or in healthy subjects during hyperglycemia [34]. A large prospective cohort study in patients with type 2 diabetes found that reduced vasodilation during flicker light stimulation is an independent risk factor for DR incidence and progression [56], and several cross-sectional studies showed that the flicker light-induced vasodilation of retinal vessels further decreases with increasing stages of DR [37, 41, 42]. Hence, neurovascular coupling may deteriorate with advancement of the disease. Our results indicate that neurodegeneration may be involved in this worsening.

Similar to findings in human patients, flicker light-induced vasodilation is early reduced in animal models of diabetes [57, 58]. In diabetic rats upregulation of inducible nitric oxide (NO) synthase (iNOS) increased retinal levels of NO [57], which. have also shown neurotoxic effects on retinal ganglion cells [59]. Inhibition of iNOS restored impaired neurovascular coupling in an animal model [57], and also improved the flicker light response in retinal veins in diabetes patients without retinopathy [60]. Several medical treatments have shown potential in improvement of neurovascular coupling [61–64]. However, following from our results the chance to restore this impairment may be higher if neurodegeneration has not yet occurred.

It is long known, that ocular stimulation with flickering light results in a reactive increase of retinal vessel diameters and retinal blood flow [65, 66]. Although the mechanisms behind this functional hyperemia are not yet known in complete detail, there is a general agreement that it is induced by increased neural activity in retinal ganglion cells, and ensures adequate supply of nutrients and metabolites to the activated neuroretinal tissue with increased metabolic demand [33]. Whereas it was traditionally assumed that blood supply in neural tissue is strictly controlled by the local energy demand, recent findings show that this rapid regulatory process is the result of a tight interaction of neurons, glia cells and pericytes, usually referred to as the neurovascular unit or complex [67].

Glia cells directly transmit light-evoked vasomotor responses from retinal neurons to capillaries without neuronal intermediates [25]. Hence, functional hyperemia is absent when the connection of neurons to glia is interrupted [25]. Diabetes disturbs the normal interaction between glia cells, retinal ganglion cells and capillaries very early on a functional level [68], and glial degeneration is followed by hypoxia and retinal ganglion cell loss [69, 70], which may then also contribute to the worsening of vascular autoregulation in the diabetic retina.

Pericytes also play a role in the regulation of retinal capillary perfusion. They can constrict and dilate capillaries independently from arterioles [71]. This makes them crucial in the regulation of retinal blood flow, because retinal capillaries form the largest volumetric portion of this vascular system. Pericyte loss is among the earliest histologic signs of diabetic retinopathy [72]. But already before pericytes are lost, damage to gap junctions, and most likely also to the recently detected interpericyte tunneling nanotubes [73, 74] lead to a loss of coordinated light-evoked vasomotor response [75–77]. Pericyte damage-induced neurovascular impairment in diabetes is therefore very likely and may additionally disturb the blood supply to retinal ganglion cells, thus contributing to their functional impairment and sensitization to degenerate on the long term.

Besides the neurodegenerative component, we also detected an association of worse glycemic control in diabetes estimated by HbA1c levels with the reduced vascular response to flicker-light stimulation, whereas age showed less influence, since it lost its significant association in the multiple regression model. The observed negative correlation with HbA1c goes in line with the results from the large population-based Maastricht Study [78]. This study also reported a negative correlation of glucose levels and flicker light response, which we did not observe. Chronic hyperglycemia in diabetes may initiate alterations in the retinal vasculature including capillary basal lamina thickening, pericyte loss and capillary non-perfusion [67, 79], as well as dilation and endothelial dysfunction in larger vessels [80, 81]. These changes could be more prominent in patients with continuously higher glucose levels reflected by higher HbA1c, and also impair vascular reactivity. However, previous studies showed that the diminished retinal vasodilation response in diabetes is not solely due to endothelial dysfunction or pre-dilation of retinal veins [81, 82]. Consistent with this, the baseline vessel calibers in the current study were not different between patients and controls, and showed no influence on vessel reactivity. In addition, chronic hyperglycemia also induces oxidative stress and low-grade inflammation which are considered to play an important role in the development of DR [50, 83], and could also contribute to neurodegenerative changes.

There was considerable variability of the vasodilation response also among healthy individuals but no significant association with GCL thickness, age or other assessed parameters. Still, higher age variability in combination with a smaller sample size may have induced this observation [84].

Our study has some limitations that need to be considered: first, this is a cross-sectional study in a relatively small cohort. Future studies in larger cohorts are needed to validate our initial results. These studies should also include a full range of diabetic retinopathy stages and perhaps diabetic maculopathy to investigate if neurovascular coupling is progressively impaired together with further increasing changes in retinal layer structure. Although not statistically significant, diabetes patients were slightly older than healthy controls, and patients with moderate DR had a longer disease duration on average, which both could have introduced some degree of measurement bias. The inclusion of age, disease duration and arterial hypertension as predictors in the multiple regression model aimed to control for possible bias. In addition, because we did not include patients without clinical DR, the detected correlations may not be directly applicable to eyes without DR, where neurodegeneration may be less or absent. In diabetes patients without neuroretinopathy, neurovascular coupling impairment may be primarily induced by other mechanisms as explained above. However, our data correspond well to previous observations that independently found either more reduction in GCL thickness [12, 49] or a further decline in functional hyperemia [37, 41, 42] in advancing stages of DR. Our study results are limited to morphological changes in retinal layers, which also include other cell types than neuronal cells. As discussed above, disturbance and degeneration of the neurovascular complex in diabetes seems to affect all different components already early on. Although degeneration of retinal ganglion cells should logically result in reduced inner neuronal activity, *direct* consequences of retinal ganglion cell loss on neurovascular coupling in patients with DR remain to be shown. The current evidence from experimental studies indicates that ganglion cell impairment and loss rather happens consequently to vascular dysfunction. Longitudinal studies investigating the vascular, neuronal and glial components of the neurovascular complex in detail, could provide more information about the underlying pathways of changes in perfusion regulation during progression of DR. Future studies should also aim at identifying additional factors that contribute to degeneration of the neurovascular complex in diabetes in order to distinguish possible treatment targets. Improvement of glycemic control, reflected by lower HbA1c, may be an appropriate strategy to restore neurovascular coupling in diabetes patients. However, our results imply that this effect is limited by the degree of retinal neurodegeneration. This should be also considered in future trials assessing the effectiveness of therapeutic agents specifically addressing neurovascular dysfunction.

In conclusion, our current results corroborate the hypothesis that the reduced reactivity of retinal vessels to light stimulation in diabetes involves an independent regulatory impairment of the retinal neurovascular complex. Furthermore, GCL thinning indicating structural degeneration in the neurovascular complex of the inner retina might contribute to the advancing disturbance of neurovascular coupling in clinically established DR, in addition to the previously observed role of chronic hyperglycemia. Because the correlation between GCL thickness with neurovascular coupling was not observed in healthy individuals, it may be specific to diabetes.

## References

[1] Barber AJ, Gardner TW, Abcouwer SF (2011) The significance of vascular and neural apoptosis to the pathology of diabetic retinopathy. Invest Ophthalmol Vis Sci 52(2):1156–1163. 10.1167/iovs.10-6293 21357409 10.1167/iovs.10-6293 PMC3053099

[2] Biallosterski C, van Velthoven ME, Michels RP, Schlingemann RO, DeVries JH, Verbraak FD (2007) Decreased optical coherence tomography-measured pericentral retinal thickness in patients with diabetes mellitus type 1 with minimal diabetic retinopathy. Br J Ophthalmol 91(9):1135–1138. 10.1136/bjo.2006.111534 17383994 10.1136/bjo.2006.111534 PMC1954913

[3] Oshitari T, Hanawa K, Adachi-Usami E (2009) Changes of macular and RNFL thicknesses measured by Stratus OCT in patients with early stage diabetes. Eye 23(4):884–889. 10.1038/eye.2008.119 18437178 10.1038/eye.2008.119

[4] Simó R, Hernández C, European Consortium for the Early Treatment of Diabetic Retinopathy (EUROCONDOR) (2012) Neurodegeneration is an early event in diabetic retinopathy: therapeutic implications. Br J Ophthalmol 96(10):1285–1290. 10.1136/bjophthalmol-2012-302005 22887976 10.1136/bjophthalmol-2012-302005

[5] De Clerck EE, Schouten JS, Berendschot TT, Kessels AG, Nuijts RM, Beckers HJ, Schram MT, Stehouwer CD, Webers CA (2015) New ophthalmologic imaging techniques for detection and monitoring of neurodegenerative changes in diabetes: a systematic review. Lancet Diabetes Endocrinol 3(8):653–663. 10.1016/S2213-8587(15)00136-9 26184671 10.1016/S2213-8587(15)00136-9

[6] Sohn EH, van Dijk HW, Jiao C, Kok PH, Jeong W, Demirkaya N, Garmager A, Wit F, Kucukevcilioglu M, van Velthoven ME, DeVries JH, Mullins RF, Kuehn MH, Schlingemann RO, Sonka M, Verbraak FD, Abràmoff MD (2016) Retinal neurodegeneration may precede microvascular changes characteristic of diabetic retinopathy in diabetes mellitus. Proc Natl Acad Sci U S A 113(19):E2655–2664. 10.1073/pnas.1522014113 27114552 10.1073/pnas.1522014113 PMC4868487

[7] van Dijk HW, Kok PH, Garvin M, Sonka M, Devries JH, Michels RP, van Velthoven ME, Schlingemann RO, Verbraak FD, Abràmoff MD (2009) Selective loss of inner retinal layer thickness in type 1 diabetic patients with minimal diabetic retinopathy. Invest Ophthalmol Vis Sci 50(7):3404–3409. 10.1167/iovs.08-3143 19151397 10.1167/iovs.08-3143 PMC2937215

[8] Cabrera DeBuc D, Somfai GM (2010) Early detection of retinal thickness changes in diabetes using Optical Coherence Tomography. Med Sci Monit 16(3):MT15–MT21 20190693

[9] van Dijk HW, Verbraak FD, Kok PH, Stehouwer M, Garvin MK, Sonka M, DeVries JH, Schlingemann RO, Abràmoff MD (2012) Early neurodegeneration in the retina of type 2 diabetic patients. Invest Ophthalmol Vis Sci 53(6):2715–2719. 10.1167/iovs.11-8997 22427582 10.1167/iovs.11-8997 PMC3366721

[10] Chhablani J, Sharma A, Goud A, Peguda HK, Rao HL, Begum VU, Barteselli G (2015) Neurodegeneration in Type 2 Diabetes: Evidence From Spectral-Domain Optical Coherence Tomography. Invest Ophthalmol Vis Sci 56(11):6333–6338. https://doi.org10.1167/iovs.15-17334 10.1167/iovs.15-17334 26436886

[11] Spaide RF (2019) Measurable aspects of the retinal neurovascular unit in diabetes, Glaucoma, and controls. Am J Ophthalmol 207:395–409. 10.1016/j.ajo.2019.04.035 31078537 10.1016/j.ajo.2019.04.035

[12] Ng DS, Chiang PP, Tan G, Cheung CG, Cheng CY, Cheung CY, Wong TY, Lamoureux EL, Ikram MK (2016) Retinal ganglion cell neuronal damage in diabetes and diabetic retinopathy. Clin Exp Ophthalmol 44(4):243–250. 10.1111/ceo.12724 26872562 10.1111/ceo.12724

[13] Kim K, Kim ES, Kim DG, Yu SY (2019) Progressive retinal neurodegeneration and microvascular change in diabetic retinopathy: longitudinal study using OCT angiography. Acta Diabetol 56(12):1275–1282. 10.1007/s00592-019-01395-6 31401734 10.1007/s00592-019-01395-6

[14] Caputo S, Di Leo MA, Falsini B, Ghirlanda G, Porciatti V, Minella A, Greco AV (1990) Evidence for early impairment of macular function with pattern ERG in type I diabetic patients. Diabetes Care 13(4):412–418. 10.2337/diacare.13.4.412 2318101 10.2337/diacare.13.4.412

[15] Juen S, Kieselbach GF (1990) Electrophysiological changes in juvenile diabetics without retinopathy. Arch Ophthalmol 108(3):372–375. 10.1001/archopht.1990.01070050070033 2310337 10.1001/archopht.1990.01070050070033

[16] Palmowski AM, Sutter EE, Bearse MA Jr, Fung W (1997) Mapping of retinal function in diabetic retinopathy using the multifocal electroretinogram. Invest Ophthalmol Vis Sci 38(12):2586–2596 9375578

[17] Lopes de Faria JM, Katsumi O, Cagliero E, Nathan D, Hirose T (2001) Neurovisual abnormalities preceding the retinopathy in patients with long-term type 1 diabetes mellitus. Graefes Arch Clin Exp Ophthalmol 239(9):643–648. 10.1007/s004170100268 11688662 10.1007/s004170100268

[18] Realini T, Lai MQ, Barber L (2004) Impact of diabetes on glaucoma screening using frequency-doubling perimetry. Ophthalmology 111(11):2133–2136. 10.1016/j.ophtha.2004.05.024 15522382 10.1016/j.ophtha.2004.05.024

[19] Stavrou EP, Wood JM (2005) Central visual field changes using flicker perimetry in type 2 diabetes mellitus. Acta Ophthalmol Scand 83(5):574–580. 10.1111/j.1600-0420.2005.00527.x 16187995 10.1111/j.1600-0420.2005.00527.x

[20] Gualtieri M, Bandeira M, Hamer RD, Damico FM, Moura AL, Ventura DF (2011) Contrast sensitivity mediated by inferred magno- and parvocellular pathways in type 2 diabetics with and without nonproliferative retinopathy. Invest Ophthalmol Vis Sci 52(2):1151–1155. 10.1167/iovs.09-3705 21051718 10.1167/iovs.09-3705

[21] Jackson GR, Scott IU, Quillen DA, Walter LE, Gardner TW (2012) Inner retinal visual dysfunction is a sensitive marker of non-proliferative diabetic retinopathy. Br J Ophthalmol 96(5):699–703. 10.1136/bjophthalmol-2011-300467 22174096 10.1136/bjophthalmol-2011-300467

[22] Zeng Y, Cao D, Yu H, Yang D, Zhuang X, Hu Y, Li J, Yang J, Wu Q, Liu B, Zhang L (2019) Early retinal neurovascular impairment in patients with diabetes without clinically detectable retinopathy. Br J Ophthalmol 103(12):1747–1752. 10.1136/bjophthalmol-2018-313582 30674454 10.1136/bjophthalmol-2018-313582

[23] Arend O, Wolf S, Remky A, Sponsel WE, Harris A, Bertram B, Reim M (1994) Perifoveal microcirculation with non-insulin-dependent diabetes mellitus. Graefes Arch Clin Exp Ophthalmol 232(4):225–231. 10.1007/BF00184010 8034211 10.1007/BF00184010

[24] Chua J, Sim R, Tan B, Wong D, Yao X, Liu X, Ting DSW, Schmidl D, Ang M, Garhöfer G, Schmetterer L (2020) Optical coherence Tomography Angiography in Diabetes and Diabetic Retinopathy. J Clin Med 9(6):1723. 10.3390/jcm9061723 32503234 10.3390/jcm9061723 PMC7357089

[25] Li X, Xie J, Zhang L, Cui Y, Zhang G, Chen X, Wang J, Zhang A, Huang T, Meng Q (2020) Identifying Microvascular and neural parameters related to the severity of Diabetic Retinopathy using Optical Coherence Tomography Angiography. Invest Ophthalmol Vis Sci 61(5):39. 10.1167/iovs.61.5.39 32441757 10.1167/iovs.61.5.39 PMC7405728

[26] Marques IP, Ferreira S, Santos T, Madeira MH, Santos AR, Mendes L, Lobo C, Cunha-Vaz J (2022) Association between Neurodegeneration and Macular Perfusion in the progression of Diabetic Retinopathy: a 3-Year longitudinal study. Ophthalmologica 245(4):335–341. 10.1159/000522527 35158351 10.1159/000522527 PMC9393829

[27] Sung JY, Lee MW, Lim HB, Ryu CK, Yu HY, Kim JY (2022) The Ganglion Cell-Inner Plexiform Layer Thickness/Vessel density of superficial vascular plexus ratio according to the progression of Diabetic Retinopathy. Invest Ophthalmol Vis Sci 63(6):4. 10.1167/iovs.63.6.4 35653120 10.1167/iovs.63.6.4 PMC9172016

[28] Sakata K, Funatsu H, Harino S, Noma H, Hori S (2006) Relationship between macular microcirculation and progression of diabetic macular edema. Ophthalmology 113(8):1385–1391. 10.1016/j.ophtha.2006.04.023 16877077 10.1016/j.ophtha.2006.04.023

[29] Palochak CMA, Lee HE, Song J, Geng A, Linsenmeier RA, Burns SA, Fawzi AA (2019) Retinal blood velocity and Flow in Early Diabetes and Diabetic Retinopathy using adaptive Optics scanning laser Ophthalmoscopy. J Clin Med 8(8):1165. 10.3390/jcm8081165 31382617 10.3390/jcm8081165 PMC6723736

[30] Pournaras CJ, Rungger-Brändle E, Riva CE, Hardarson SH, Stefansson E (2008) Regulation of retinal blood flow in health and disease. Prog Retin Eye Res 27(3):284–330. 10.1016/j.preteyeres.2008.02.002 18448380 10.1016/j.preteyeres.2008.02.002

[31] Metea MR, Newman EA (2007) Signalling within the neurovascular unit in the mammalian retina. Exp Physiol 92(4):635–640. 10.1113/expphysiol.2006.036376 17434916 10.1113/expphysiol.2006.036376 PMC2279186

[32] Andreone BJ, Lacoste B, Gu C (2015) Neuronal and vascular interactions. Annu Rev Neurosci 38:25–46. 10.1146/annurev-neuro-071714-033835 25782970 10.1146/annurev-neuro-071714-033835 PMC5729758

[33] Garhöfer G, Chua J, Tan B, Wong D, Schmidl D, Schmetterer L (2020) Retinal neurovascular coupling in diabetes. J Clin Med 9(9):2829. 10.3390/jcm9092829 32882896 10.3390/jcm9092829 PMC7565465

[34] Dorner GT, Garhöfer G, Huemer KH, Riva CE, Wolzt M, Schmetterer L (2003) Hyperglycemia affects flicker-induced vasodilation in the retina of healthy subjects. Vis Res 43(13):1495–1500. 10.1016/s0042-6989(03)00170-6 12767316 10.1016/s0042-6989(03)00170-6

[35] Kwan CC, Lee HE, Schwartz G, Fawzi AA (2020) Acute Hyperglycemia reverses neurovascular Coupling during Dark to Light Adaptation in healthy subjects on Optical Coherence Tomography Angiography. Invest Ophthalmol Vis Sci 61(4):38. 10.1167/iovs.61.4.38 32340033 10.1167/iovs.61.4.38 PMC7401911

[36] Garhöfer G, Zawinka C, Resch H, Kothy P, Schmetterer L, Dorner GT (2004) Reduced response of retinal vessel diameters to flicker stimulation in patients with diabetes. Br J Ophthalmol 88(7):887–891. 10.1136/bjo.2003.033548 15205231 10.1136/bjo.2003.033548 PMC1772243

[37] Mandecka A, Dawczynski J, Blum M, Müller N, Kloos C, Wolf G, Vilser W, Hoyer H, Müller UA (2007) Influence of flickering light on the retinal vessels in diabetic patients. Diabetes Care 30(12):3048–3052. 10.2337/dc07-0927 17728481 10.2337/dc07-0927

[38] Zhang YS, Mucollari I, Kwan CC, Dingillo G, Amar J, Schwartz GW, Fawzi AA (2020) Reversed neurovascular coupling on Optical Coherence Tomography Angiography is the earliest detectable abnormality before Clinical Diabetic Retinopathy. J Clin Med 9(11):3523. 10.3390/jcm9113523 33142724 10.3390/jcm9113523 PMC7692675

[39] Lecleire-Collet A, Audo I, Aout M, Girmens JF, Sofroni R, Erginay A, Le Gargasson JF, Mohand-Saïd S, Meas T, Guillausseau PJ, Vicaut E, Paques M, Massin P (2011) Evaluation of retinal function and flicker light-induced retinal vascular response in normotensive patients with diabetes without retinopathy. Invest Ophthalmol Vis Sci 52(6):2861–2867. 10.1167/iovs.10-5960 21282578 10.1167/iovs.10-5960

[40] Lasta M, Pemp B, Schmidl D, Boltz A, Kaya S, Palkovits S, Werkmeister R, Howorka K, Popa-Cherecheanu A, Garhöfer G, Schmetterer L (2013) Neurovascular dysfunction precedes neural dysfunction in the retina of patients with type 1 diabetes. Invest Ophthalmol Vis Sci 54(1):842–847. 10.1167/iovs.12-10873 23307962 10.1167/iovs.12-10873

[41] Nguyen TT, Kawasaki R, Wang JJ, Kreis AJ, Shaw J, Vilser W, Wong TY (2009) Flicker light-induced retinal vasodilation in diabetes and diabetic retinopathy. Diabetes Care 32(11):2075–2080. 10.2337/dc09-0075 19641162 10.2337/dc09-0075 PMC2768208

[42] Hommer N, Kallab M, Schlatter A, Janku P, Werkmeister RM, Howorka K, Schmidl D, Schmetterer L, Garhöfer G (2022) Neuro-vascular coupling and heart rate variability in patients with type II diabetes at different stages of diabetic retinopathy. Front Med 9:1025853. 10.3389/fmed.2022.1025853 10.3389/fmed.2022.1025853 PMC9684184 36438055

[43] Early Treatment Diabetic Retinopathy Study Research Group (1991) Grading diabetic retinopathy from stereoscopic color fundus photographs–an extension of the modified Airlie House classification. ETDRS report number 10. Ophthalmology 98(5 Suppl):786–806. 10.1016/S0161-6420(13)38012-9 2062513

[44] Garhofer G, Bek T, Boehm AG, Gherghel D, Grunwald J, Jeppesen P, Kergoat H, Kotliar K, Lanzl I, Lovasik JV, Nagel E, Vilser W, Orgul S, Schmetterer L, Ocular Blood Flow Research Association (2010) Use of the retinal vessel analyzer in ocular blood flow research. Acta Ophthalmol 88(7):717–722. 10.1111/j.1755-3768.2009.01587.x 19681764 10.1111/j.1755-3768.2009.01587.x

[45] Castro Lima V, Rodrigues EB, Nunes RP, Sallum JF, Farah ME, Meyer CH (2011) Simultaneous confocal scanning laser ophthalmoscopy combined with high-resolution spectral-domain optical coherence tomography: a review. J Ophthalmol 2011:743670. 10.1155/2011/743670 22132313 10.1155/2011/743670 PMC3206361

[46] Pemp B, Kardon RH, Kircher K, Pernicka E, Schmidt-Erfurth U, Reitner A (2013) Effectiveness of averaging strategies to reduce variance in retinal nerve fibre layer thickness measurements using spectral-domain optical coherence tomography. Graefes Arch Clin Exp Ophthalmol 251(7):1841–1848. 10.1007/s00417-013-2337-0 23589277 10.1007/s00417-013-2337-0

[47] Carpineto P, Toto L, Aloia R, Ciciarelli V, Borrelli E, Vitacolonna E, Di Nicola M, Di Antonio L, Mastropasqua R (2016) Neuroretinal alterations in the early stages of diabetic retinopathy in patients with type 2 diabetes mellitus. Eye 30(5):673–679. 10.1038/eye.2016.13 26869156 10.1038/eye.2016.13 PMC4869127

[48] van de Kreeke JA, Darma S, Chan Pin Yin JMPL, Tan HS, Abramoff MD, Twisk JWR, Verbraak FD (2020) The spatial relation of diabetic retinal neurodegeneration with diabetic retinopathy. PLoS ONE 15(4):e0231552. 10.1371/journal.pone.0231552 32298369 10.1371/journal.pone.0231552 PMC7161968

[49] Aschauer J, Pollreisz A, Karst S, Hülsmann M, Hajdu D, Datlinger F, Egner B, Kriechbaum K, Pablik E, Schmidt-Erfurth UM (2022) Longitudinal analysis of microvascular perfusion and neurodegenerative changes in early type 2 diabetic retinal disease. Br J Ophthalmol 106(4):528–533. 10.1136/bjophthalmol-2020-317322 33293271 10.1136/bjophthalmol-2020-317322

[50] Abu-El-Asrar AM, Dralands L, Missotten L, Al-Jadaan IA, Geboes K (2004) Expression of apoptosis markers in the retinas of human subjects with diabetes. Invest Ophthalmol Vis Sci 45(8):2760–2766. 10.1167/iovs.03-1392 15277502 10.1167/iovs.03-1392

[51] Gastinger MJ, Barber AJ, Khin SA, McRill CS, Gardner TW, Marshak DW (2001) Abnormal centrifugal axons in streptozotocin-diabetic rat retinas. Invest Ophthalmol Vis Sci 42(11):2679–2685 11581216 PMC3341734

[52] Gastinger MJ, Singh RS, Barber AJ (2006) Loss of cholinergic and dopaminergic amacrine cells in streptozotocin-diabetic rat and Ins2Akita-diabetic mouse retinas. Invest Ophthalmol Vis Sci 47(7):3143–3150. 10.1167/iovs.05-1376 16799061 10.1167/iovs.05-1376

[53] Fernandez DC, Pasquini LA, Dorfman D, Aldana Marcos HJ, Rosenstein RE (2012) Early distal axonopathy of the visual pathway in experimental diabetes. Am J Pathol 180(1):303–313. 10.1016/j.ajpath.2011.09.018 22079928 10.1016/j.ajpath.2011.09.018 PMC3244601

[54] Mandecka A, Dawczynski J, Vilser W, Blum M, Müller N, Kloos C, Wolf G, Müller UA (2009) Abnormal retinal autoregulation is detected by provoked stimulation with flicker light in well-controlled patients with type 1 diabetes without retinopathy. Diabetes Res Clin Pract 86(1):51–55. 10.1016/j.diabres.2009.06.017 19646772 10.1016/j.diabres.2009.06.017

[55] Patel SR, Bellary S, Qin L, Balanos GM, McIntyre D, Gherghel D (2012) Abnormal retinal vascular reactivity in individuals with impaired glucose tolerance: a preliminary study. Invest Ophthalmol Vis Sci 53(9):5102–5108. 10.1167/iovs.12-9512 22743316 10.1167/iovs.12-9512

[56] Lim LS, Ling LH, Ong PG, Foulds W, Tai ES, Wong TY (2017) Dynamic responses in Retinal Vessel Caliber with Flicker Light Stimulation and Risk of Diabetic Retinopathy and its progression. Invest Ophthalmol Vis Sci 58(5):2449–2455. 10.1167/iovs.16-21008 28460046 10.1167/iovs.16-21008

[57] Mishra A, Newman EA (2010) Inhibition of inducible nitric oxide synthase reverses the loss of functional hyperemia in diabetic retinopathy. Glia 58(16):1996–2004. 10.1002/glia.21068 20830810 10.1002/glia.21068 PMC3206643

[58] Hanaguri J, Yokota H, Watanabe M, Yamagami S, Kushiyama A, Kuo L, Nagaoka T (2021) Retinal blood flow dysregulation precedes neural retinal dysfunction in type 2 diabetic mice. Sci Rep 11(1):18401. 10.1038/s41598-021-97651-3 34526573 10.1038/s41598-021-97651-3 PMC8443656

[59] Kawasaki A, Otori Y, Barnstable CJ (2000) Müller cell protection of rat retinal ganglion cells from glutamate and nitric oxide neurotoxicity. Invest Ophthalmol Vis Sci 41(11):3444–3450 11006237

[60] Petersen L, Bek T (2016) Preserved pressure autoregulation but disturbed cyclo-oxygenase and nitric Oxide effects on Retinal Arterioles during Acute Hypoxia in Diabetic patients without Retinopathy. Ophthalmologica 235(2):114–120. 10.1159/000443147 26741496 10.1159/000443147

[61] Honasoge A, Nudleman E, Smith M, Rajagopal R (2019) Emerging insights and interventions for Diabetic Retinopathy. Curr Diab Rep 19(10):100. 10.1007/s11892-019-1218-2 31506830 10.1007/s11892-019-1218-2 PMC7941754

[62] Nian S, Lo ACY, Mi Y, Ren K, Yang D (2021) Neurovascular unit in diabetic retinopathy: pathophysiological roles and potential therapeutical targets. Eye Vis 8(1):15. 10.1186/s40662-021-00239-1 10.1186/s40662-021-00239-1 PMC8088070 33931128

[63] Forst T, Michelson G, Ratter F, Weber MM, Anders S, Mitry M, Wilhelm B, Pfützner A (2012) Addition of liraglutide in patients with type 2 diabetes well controlled on metformin monotherapy improves several markers of vascular function. Diabet Med 29(9):1115–1118. 10.1111/j.1464-5491.2012.03589.x 22288732 10.1111/j.1464-5491.2012.03589.x

[64] Hanaguri J, Nagai N, Yokota H, Kushiyama A, Watanabe M, Yamagami S, Nagaoka T (2022) Fenofibrate Nano-Eyedrops ameliorate Retinal Blood Flow Dysregulation and Neurovascular Coupling in type 2 Diabetic mice. Pharmaceutics 14(2):384. 10.3390/pharmaceutics14020384 35214116 10.3390/pharmaceutics14020384 PMC8876509

[65] Formaz F, Riva CE, Geiser M (1997) Diffuse luminance flicker increases retinal vessel diameter in humans. Curr Eye Res 16(12):1252–1257. 10.1076/ceyr.16.12.1252.5021 9426960 10.1076/ceyr.16.12.1252.5021

[66] Polak K, Schmetterer L, Riva CE (2002) Influence of flicker frequency on flicker-induced changes of retinal vessel diameter. Invest Ophthalmol Vis Sci 43(8):2721–2726 12147608

[67] Yang S, Zhang J, Chen L (2020) The cells involved in the pathological process of diabetic retinopathy. Biomed Pharmacother 132:110818. 10.1016/j.biopha.2020.110818 33053509 10.1016/j.biopha.2020.110818

[68] Mills SA, Jobling AI, Dixon MA, Bui BV, Vessey KA, Phipps JA, Greferath U, Venables G, Wong VHY, Wong CHY, He Z, Hui F, Young JC, Tonc J, Ivanova E, Sagdullaev BT, Fletcher EL (2021) Fractalkine-induced microglial vasoregulation occurs within the retina and is altered early in diabetic retinopathy. Proc Natl Acad Sci U S A 118(51):e2112561118. 10.1073/pnas.2112561118 34903661 10.1073/pnas.2112561118 PMC8713803

[69] Ly A, Yee P, Vessey KA, Phipps JA, Jobling AI, Fletcher EL (2011) Early inner retinal astrocyte dysfunction during diabetes and development of hypoxia, retinal stress, and neuronal functional loss. Invest Ophthalmol Vis Sci 52(13):9316–9326. 10.1167/iovs.11-7879 22110070 10.1167/iovs.11-7879

[70] Coughlin BA, Feenstra DJ, Mohr S (2017) Müller cells and diabetic retinopathy. Vis Res 139:93–100. 10.1016/j.visres.2017.03.013 28866025 10.1016/j.visres.2017.03.013 PMC5794018

[71] Hall CN, Reynell C, Gesslein B, Hamilton NB, Mishra A, Sutherland BA, O’Farrell FM, Buchan AM, Lauritzen M, Attwell D (2014) Capillary pericytes regulate cerebral blood flow in health and disease. Nature 508(7494):55–60. 10.1038/nature13165 24670647 10.1038/nature13165 PMC3976267

[72] Fletcher EL, Dixon MA, Mills SA, Jobling AI (2023) Anomalies in neurovascular coupling during early diabetes: a review. Clin Exp Ophthalmol 51(1):81–91. 10.1111/ceo.14190 36349522 10.1111/ceo.14190 PMC10947109

[73] Alarcon-Martinez L, Villafranca-Baughman D, Quintero H, Kacerovsky JB, Dotigny F, Murai KK, Prat A, Drapeau P, Di Polo A (2020) Interpericyte tunnelling nanotubes regulate neurovascular coupling. Nature 585(7823):91–95. 10.1038/s41586-020-2589-x 32788726 10.1038/s41586-020-2589-x

[74] Alarcon-Martinez L, Shiga Y, Villafranca-Baughman D, Belforte N, Quintero H (2022) Pericyte dysfunction and loss of interpericyte tunneling nanotubes promote neurovascular deficits in glaucoma. Proc Natl Acad Sci U S A 119(7):e2110329119. 10.1073/pnas.2110329119 35135877 10.1073/pnas.2110329119 PMC8851476

[75] Oku H, Kodama T, Sakagami K, Puro DG (2001) Diabetes-induced disruption of gap junction pathways within the retinal microvasculature. Invest Ophthalmol Vis Sci 42(8):1915–1920 11431461

[76] Ivanova E, Kovacs-Oller T, Sagdullaev BT (2017) Vascular pericyte impairment and Connexin43 gap Junction Deficit Contribute to Vasomotor decline in Diabetic Retinopathy. J Neurosci 37(32):7580–7594. 10.1523/JNEUROSCI.0187-17.2017 28674171 10.1523/JNEUROSCI.0187-17.2017 PMC5551058

[77] Kovacs-Oller T, Ivanova E, Bianchimano P, Sagdullaev BT (2020) The pericyte connectome: spatial precision of neurovascular coupling is driven by selective connectivity maps of pericytes and endothelial cells and is disrupted in diabetes. Cell Discov 6:39. 10.1038/s41421-020-0180-0 32566247 10.1038/s41421-020-0180-0 PMC7296038

[78] Sörensen BM, Houben AJ, Berendschot TT, Schouten JS, Kroon AA, van der Kallen CJ, Henry RM, Koster A, Sep SJ, Dagnelie PC, Schaper NC, Schram MT, Stehouwer CD (2016) Prediabetes and type 2 diabetes are Associated with generalized microvascular dysfunction: the Maastricht Study. Circulation 134(18):1339–1352. 10.1161/CIRCULATIONAHA.116.023446 27678264 10.1161/CIRCULATIONAHA.116.023446

[79] Cai J, Boulton M (2002) The pathogenesis of diabetic retinopathy: old concepts and new questions. Eye 16(3):242–260. 10.1038/sj.eye.6700133 12032713 10.1038/sj.eye.6700133

[80] Stefansson E, Landers MB 3rd, Wolbarsht ML (1983) Oxygenation and vasodilatation in relation to diabetic and other proliferative retinopathies. Ophthalmic Surg 14(3):209–226. 10.3928/1542-8877-19830301-01 6190118

[81] Pemp B, Weigert G, Karl K, Petzl U, Wolzt M, Schmetterer L, Garhofer G (2009) Correlation of flicker-induced and flow-mediated vasodilatation in patients with endothelial dysfunction and healthy volunteers. Diabetes Care 32(8):1536–1541. 10.2337/dc08-2130 19478197 10.2337/dc08-2130 PMC2713642

[82] Pemp B, Garhofer G, Weigert G, Karl K, Resch H, Wolzt M, Schmetterer L (2009) Reduced retinal vessel response to flicker stimulation but not to exogenous nitric oxide in type 1 diabetes. Invest Ophthalmol Vis Sci 50(9):4029–4032. 10.1167/iovs.08-3260 19369238 10.1167/iovs.08-3260

[83] Martins B, Amorim M, Reis F, Ambrósio AF, Fernandes R (2020) Extracellular vesicles and MicroRNA: putative role in diagnosis and treatment of Diabetic Retinopathy. Antioxidants 9(8):705. 10.3390/antiox9080705 32759750 10.3390/antiox9080705 PMC7463887

[84] Seshadri S, Ekart A, Gherghel D (2016) Ageing effect on flicker-induced diameter changes in retinal microvessels of healthy individuals. Acta Ophthalmol 94(1):e35–42. 10.1111/aos.12786 26149453 10.1111/aos.12786 PMC5034828

